# Alteration in Superoxide Dismutase 1 Causes Oxidative Stress and p38 MAPK Activation Following RVFV Infection

**DOI:** 10.1371/journal.pone.0020354

**Published:** 2011-05-31

**Authors:** Aarthi Narayanan, Taissia Popova, Michael Turell, Jessica Kidd, Jessica Chertow, Serguei G. Popov, Charles Bailey, Fatah Kashanchi, Kylene Kehn-Hall

**Affiliations:** 1 National Center for Biodefense and Infectious Diseases, George Mason University, Manassas, Virginia, United States of America; 2 Virology Division, United States Army Medical Research Institute of Infectious Diseases, Fort Detrick, Maryland, United States of America; 3 The Neurological Institute, MDA/ALS Research Center, New York, New York, United States of America; University of South Florida College of Medicine, United States of America

## Abstract

Rift Valley fever (RVF) is a zoonotic disease caused by Rift Valley fever virus (RVFV). RVFV is a category A pathogen that belongs to the genus *Phlebovirus*, family *Bunyaviridae*. Understanding early host events to an infectious exposure to RVFV will be of significant use in the development of effective therapeutics that not only control pathogen multiplication, but also contribute to cell survival. In this study, we have carried out infections of human cells with a vaccine strain (MP12) and virulent strain (ZH501) of RVFV and determined host responses to viral infection. We demonstrate that the cellular antioxidant enzyme superoxide dismutase 1 (SOD1) displays altered abundances at early time points following exposure to the virus. We show that the enzyme is down regulated in cases of both a virulent (ZH501) and a vaccine strain (MP12) exposure. Our data demonstrates that the down regulation of SOD1 is likely to be due to post transcriptional processes and may be related to up regulation of TNFα following infection. We also provide evidence for extensive oxidative stress in the MP12 infected cells. Concomitantly, there is an increase in the activation of the p38 MAPK stress response, which our earlier published study demonstrated to be an essential cell survival strategy. Our data suggests that the viral anti-apoptotic protein NSm may play a role in the regulation of the cellular p38 MAPK response. Alterations in the host protein SOD1 following RVFV infection appears to be an early event that occurs in multiple cell types. Activation of the cellular stress response p38 MAPK pathway can be observed in all cell types tested. Our data implies that maintaining oxidative homeostasis in the infected cells may play an important role in improving survival of infected cells.

## Introduction

Rift Valley fever virus (RVFV) is an arthropod borne virus that belongs to the genus *Phlebovirus*, family *Bunyaviridae* [1], [2], [3]. It is an enveloped virus with a single stranded, tripartite RNA genome. The large (L) and the medium (M) segments are of negative polarity and the small (S) segment displays ambisense polarity. The L segment codes for the RNA dependent RNA polymerase. The M segment produces the structural proteins Gn and Gc and two non structural proteins namely, the 78 kDa protein and NSm. The S segment codes for the structural nucleoprotein (N) and the nonstructural protein NSs. NSs has been demonstrated to regulate interferon response and host transcriptional shutoff early in the infectious process [4], [5], [6], [7], [8], [9], [10], [11], [12]. NSm, the other viral nonstructural protein, has been demonstrated to be anti-apoptotic in function [13], [14].

RVFV is the causative agent of Rift Valley Fever (RVF) which is a disease that primarily affects live stock, manifested as fevers and cases of spontaneous abortions in adult animals and high mortality in young animals [15], [16]. The disease can be spread to humans by mosquitoes, mainly by members of the genera Aedes and Culex [17]. There have been several recent outbreaks of RVF reported in different parts of the globe including Kenya [18], [19], Saudi Arabia [20] and Yemen [21] with significantly high fatality rates. In humans, the virus can cause disease with a range of severities. In most cases, the patients develop a mild illness with fever, headache, myalgia and liver abnormalities. In a small percentage of the cases, the illness can progress to hemorrhagic fever or meningoencephalitis. In addition, ocular sequellae can occur that cause retinal damage, including blindness. About 1% of the affected humans die of the disease although, in recent years this percentage has increased (closer to 45%), probably due to increased incidence of people seeking medical attention. RVFV is classified as both an emerging infectious agent and as a category A Biodefense pathogen. While Ribavarin is used in some cases as a therapeutic, there are undesirable side effects and alternative, effective therapeutics are needed. One of the recurrent themes in recent studies of many infectious diseases is therapeutic approach by modulation of host response elicited due to an infectious exposure.

Oxidative stress due to viral infection has been recognized to be an important contributor to pathogenesis in many cases such as hepatitis B, hepatitis C and Dengue infection [22], [23], [24]. The cell has extensive machinery to ensure maintenance of oxidative homeostasis. One important component of the cellular antioxidant machinery is the superoxide dismutase (SOD) family of enzymes [25]. SOD1 is the most abundant cytoplasmic antioxidant protein while SOD2 is a mitochondrial enzyme. Disruption of the host antioxidant machinery is associated with many disease states. Functionally altered SOD1 has been associated with a metabolic disorder called Amyotropic Lateral Sclerosis (ALS) [26]. ALS accounts for many motor neuron disorders and is a progressive and fatal neurodegenerative disease leading to paralysis of skeletal muscles and premature cell death.

The redox status of a given cell plays a vital role in regulating the activity of multiple transcription factors and activators such as NFkB, AP1 and p53 and hence influences cellular target gene expression and modulates multiple cellular signaling pathways. Maintaining appropriate levels of reactive oxygen species (ROS) is necessary for normal physiological functioning of cells [27]. Our recently published phosphoproteomic study has revealed that many of the transcription factors that are subject to modulation by ROS are altered following RVFV infection [28]. Therefore, we wanted to determine if RVFV infection caused oxidative stress in infected human cells and if there were host responses associated with such a cellular stress condition. Our results demonstrate that viral infection causes an early decrease in SOD1 and there is significant oxidative stress in infected cells. Our results also show that the down regulation of SOD1 is accompanied by increased phosphorylation of p38 MAPK, which we believe is a protective response that is necessary to delay onset of apoptosis in the infected cells.

## Methods

### Cell culture, viral infection and extract preparation

Human small airway lung epithelial cells (HSAECs) were grown in Ham's F12 medium as per vendor's protocol. Ham's F12 was supplemented with 0.4% nonessential amino acids, 1% pyruvate, 0.2% β-mercaptoethanol and 10% FBS. For experiments using RVFV MP12 strain, 10^6^ HSAECs were cultured in 6-well plates. Cultured cells were infected with MP12 or the NSm mutant (ΔNSm) (MOI of 3) by overlaying a suspension of virus in media on the cells and incubating them for an hour at 37°C in the presence of 5% CO_2_. For infection with RVFV ZH501, 10^6^ cells per well were grown in 6-well plates. To carry out the infection, the growth media was removed, the cells washed with phosphate buffered saline (PBS), and 100 µl of either a RVFV suspension (MOI of 0.002) in diluent (10% heat-inactivated FBS in Medium 199 with Earle's salts, NaHCO_3_, and antibiotics) or 100 µl of diluent added to each well. Following one hour incubation at 35.5°C, 3 ml of the supplemented Ham's F12 culture media was added to each well and the cells maintained at 35.5°C. At 24, 30, 48 and 72 h post infection, and from unexposed wells at time 0, supernatant was removed from the wells, the cells washed with 1.5 ml of phosphate PBS, and cells collected in 500 µl of lysis buffer [1∶1 mixture of T-PER reagent (Pierce, IL), 2× Tris-glycine SDS sample buffer (Novex, Invitrogen), 33 mM DTT, and protease and phosphatase inhibitor cocktail (1× Halt cocktail, Pierce)] and boiled for 10 min. HepG2 cells and 293T cells were cultured using standard protocols. MP12 infections were carried out as described for HSAECs.

### Western blot analysis

Ten to twenty microliters of cell lysates were separated on either 4–12% Bis-Tris gels or 4–20% Tris-glycine gels and transferred to nitrocellulose membranes using iBlot Gel Transfer apparatus (Invitrogen). The membranes were blocked with 3% dry milk solution in PBS-T for an hour at room temperature. Primary antibodies to RVFV (Cat# 4519, ProSci), SOD1 (Cat# ab16831, Abcam), SOD2 (Cat# ab13533, Abcam), HRP conjugated Actin (Cat# ab49900-100, Abcam), VEGF-receptor (Cat# 2478S, Cell signaling), MKK3/6 (Cat# 9231S, Cell signaling), phospho-p38 (Cat# 9211S, Cell signaling), phospho-Hsp27-Ser82 (Cat# 2401S, Cell signaling), p38 (Cat#9212, Cell signaling) and Hsp27 (Cat#2402, Cell signaling) were diluted in 3% milk solution at 1∶1000 and incubated overnight at 4°C. The blots were then washed 3 times with PBS-T and incubated with secondary HRP-coupled goat anti-rabbit and anti-mouse antibody diluted 1∶10,000 in 3% milk. The blots were visualized by chemiluminescence using SuperSignal West Femto Maximum Sensitivity Substrate kit (ThermoScientific) and a Bio Rad Molecular Imager ChemiDoc XRS system (Bio-Rad). The band intensities were calculated using Quantity One 4.6.5 software (Bio Rad).

### RT-PCR analyses

One million cells were seeded in 6-well plates and grown to confluency. Total RNA from infected cells were obtained using Qiagen's RNeasy Mini RNA isolation kit following manufacturer's instructions. Total RNA yield was quantified using the Ribogreen RNA quantitation kit (Molecular Probes) and equivalent amount of RNA from each sample was used in cDNA synthesis reactions. cDNA was synthesized using Superscript Reverse Transcriptase cDNA kit (Invitrogen) following manufacturer's protocol. PCR was carried out for 35 cycles using specific primers to human TNFα (5′ CTA TCT GGG AGG GGT CTT CC 3′; 5′ GGT TGA GGG TGT CTG AAG GA 3′), GAPDH (5′ CAT CAC CAT CTT CCA GGA GC 3′; 5′ GGA TGA TGT TCT GGA GAG CC 3′) and SOD1 (5′ GAA GGT GTG GGG AAG CAT TA 3′; 5′ ACA TTG CCC AAG TCT CCA AC 3′) by standard methods. Ethidium bromide stained gels were visualized using the Bio Rad Molecular Imager ChemiDoc XRS system (Bio Rad). The band intensities were calculated using Quantity One 4.6.5 software (Bio Rad). For quantitative-RT-PCR experiments, cDNA was amplified in triplicate (40 cycles) using Power SYBR® Green PCR Master Mix (Invitrogen). Reactions were analyzed on an ABI 7000 and fold changes calculated using the relative standard curve method.

### Measurement of Oxidative stress

Mitosox Red superoxide indicator was used to measure ROS as per manufacturer's (Molecular Probes) instructions. Briefly, cells were incubated with Mitosox reagent for 30 min at 37°C. Following incubation, slides were washed with HBSS, fixed with paraformaldehyde and stained with DAPI to visualize nuclei. Fluorescence microscopy was carried out using a Nikon Eclipse 90i microscope and quantification of fluorescence was carried out using Nikon Elements software.

### TNFα treatment of HSAECs

One million cells were seeded in 6-well plates and grown to confluency. Cells were maintained in serum free Ham's F12 medium for 48 h. Ten, fifty and one hundred ng/ml of TNFα was added drop-wise onto the media and cells were incubated at 37°C with 5% CO_2_. Whole cell extracts were prepared in lysis buffer following TNFα treatment for 4 and 24 h as indicated above and boiled for 10 min immediately after lysis prior to processing for western blots.

### siRNA transfection

One million HSAECs were seeded in 6-well plates and grown to confluency overnight in antibiotic free Ham's F12 media. The next day, siRNA against human SOD1 (500 nM, Dharmacon) was transfected using Lipofectamine as per manufacturer's instructions. siRNA against luciferase was also purchased from Dharmacon for use as negative control. Twenty-four h post transfection, the medium was changed to regular Ham's F12 with antibiotics, but serum free and maintained for 96 h before use in flow cytometry analysis.

### Flow cytometry

Transfected cells were prepared for flow cytometry analysis by first collecting the supernatants from the wells and spinning them at 2000 rpm for 10 min in a refrigerated microcentrifuge. The cells were washed twice in 1× PBS (without calcium and magnesium) and trypsinized. The trypsin was neutralized by adding back cold media with 10% serum and the cells were spun down as indicated above. The cell pellet was washed twice with 1× PBS and resuspended in 70% ice cold ethanol. The cells were rehydrated using 1× PBS (without calcium and magnesium) for at least 15 min and spun down as described. The cells were stained with 1 ml of staining solution with propidium iodide staining solution and analyzed using BD FacsCalibur flow cytometer and CELLQuest software (BD Biosciences, Bedford, MA, USA) [29], [30], [31].

### Statistical analyses

All quantifications are based on data obtained from triplicate samples unless indicated otherwise. Error bars in all the figures indicate standard error in a two-tailed t-test. P values were calculated using paired student's t-test.

## Results

### The host cell antioxidant protein SOD1 is down regulated following exposure to MP12 strain of RVFV

**Figure 1:**
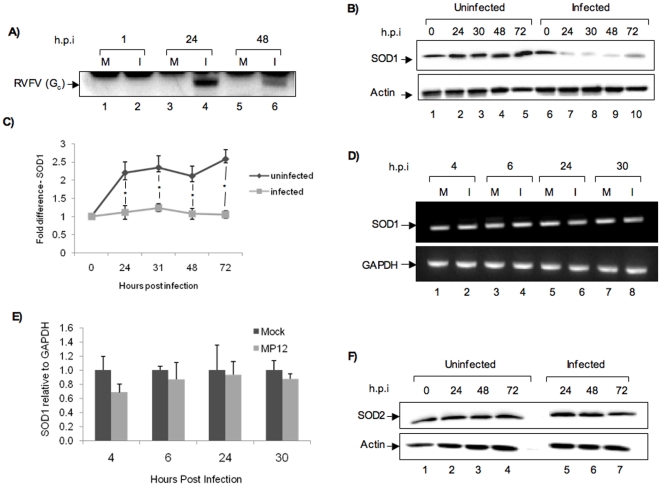
Alterations in SOD1 protein following infection by MP12 virus. A) Viral infection was confirmed by western blot analysis of HSAEC extracts using anti-RVFV antibody. B) HSAECs infected with MP12 virus (MOI of 3) were harvested at 24, 30, 48 and 72 h post infection. Cell extracts were analyzed by western blot using anti-SOD1 antibody. Actin was utilized as a loading control. C) Quantification of fold differences in SOD1 band intensities between uninfected control cells and MP12 infected cells over the time course of infection is indicated. The quantification is representative of three independent experiments. * indicates p≤0.01. D) Total RNA was obtained from HSAECs infected with MP12 virus at 4, 6, 24 and 30 h post infection and analyzed by RT-PCR with primers against SOD1. GAPDH was used as a loading control. M indicates mock-infected cells and I indicates MP12-infected cells. E) Total RNA from MP12 and mock infected samples were analyzed by q-RT-PCR with primers to SOD1. Data was normalized to GAPDH RNA levels. The results include data from three experiments. F) MP12-infected HSAEC extracts were analyzed by western blot using anti-SOD2 antibody. Actin was used as a loading control.

Oxidative stress resulting from viral infections is a well characterized phenomenon. Our phosphoproteomic analysis of RVFV infected cells revealed that multiple signaling and transcription factors that are subject to regulation by ROS were altered after infection [28]. So, we wished to determine if exposure of human cells to RVFV would result in significant oxidative stress in the infected cell. We exposed human lung epithelial cells to the MP12 strain of RVFV and first evaluated the levels of the two major enzymes involved in the maintenance of cellular oxidative homeostasis, SOD1 and SOD2 at multiple time points after infection. Whole cell extracts were obtained at 0, 24, 30, 48 and 72 h post infection and were analyzed by western blot analysis for changes in SOD1 (Figure 1B). We observed that in comparison with uninfected cells, the infected cells showed a consistent decrease in SOD1 at early time points (24 and 30 h) (Figure 1B and C). We confirmed viral infection in these cells by analyzing for viral proteins by western blots using anti-RVFV antibody (Figure 1A). We then asked whether this reduced protein levels at early time points was due to transcriptional or post transcriptional events. To answer that question, we carried out RT-PCR studies with primers specific to SOD1. GAPDH expression levels were analyzed as a control. We did not find any striking decrease in the SOD1 expression levels (Figure 1D) suggesting that the alteration in protein level is likely to be post transcriptional. In order to sensitively evaluate whether there is any transcriptional down regulation of SOD1, we carried out additional quantitative RT-PCR (q-RT-PCR) analyses with SOD1 and GAPDH primers. Our q-RT-PCR studies confirmed our RT-PCR analysis (Figure 1E) that no significant changes (p-values>0.5) in SOD1 transcripts could be observed in the infected samples. Next, we carried out western blot analyses with antibodies to superoxide dismutase 2 (SOD2) and found that SOD2 did not display early changes in protein levels (Figure 1F). Collectively, our data indicates that following exposure to MP12, human cells display a lowered abundance of the host antioxidant protein SOD1 and the down regulation appears to be due to post transcriptional effects.

### Human cells experience oxidative stress following MP12 exposure

**Figure 2:**
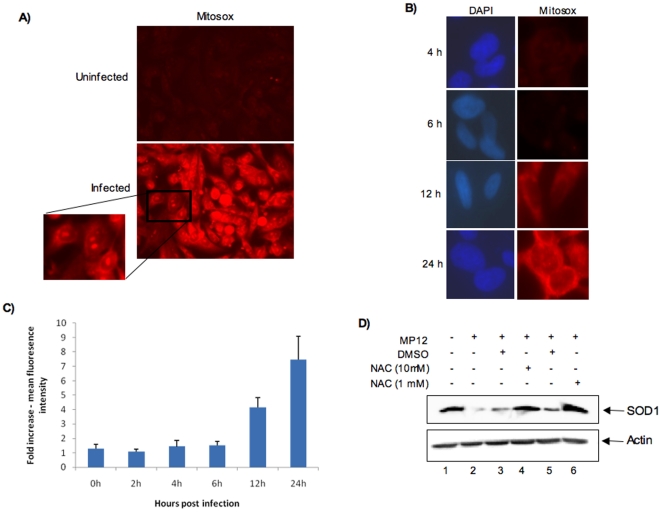
Oxidative stress in cells infected with MP12 strain of RVFV. A) HSAECs were infected with MP12 virus (MOI of 3) and 24 h later stained with 3 µg/ml of Mitosox reagent. After incubation for 30 min the Mitosox reagent was removed, cells washed 3× with HBSS, fixed with paraformaldehyde, visualized. Boxed and magnified inset shows Mitosox staining inside nucleoli. B) MP12 infected HSAECs were stained with Mitosox reagent at 4, 6, 12 and 24 h post infection and visualized as described earlier. C) Mean fluorescence intensity was calculated by averaging flouresence in ten fields picked randomly from quadruplicate samples for every time point. The mean fluorescence intensity of the infected samples was then calculated as fold difference over uninfected cells obtained at comparable time points. * p<0.00005. D) HSAECs were pre- and post treated with DMSO or increasing concentrations of NAC. Untreated and treated cells were infected with MP12 virus and SOD1 protein levels were analyzed by western blot with SOD1 antibody. Actin was used as a control.

Our observation that SOD1 shows a decrease at early time points following infection prompted us to ask the direct question whether infected cells experience oxidative stress at comparable time points. To address that question, we performed Mitosox staining of the infected cells at 24 h post infection. Mitosox is a recently validated fluorogenic dye that is used for sensitive detection of superoxide in cells [32], [33], [34]. Mitosox is a cell permeant dye that is rapidly and selectively targeted to mitochondria, an important intracellular source of ROS. It is oxidized by accumulating superoxide and emits a red fluorescence. Increasing fluorescence intensity is hence indicative of accumulating ROS, which in turn points to defects in oxidative homeostasis. We measured fluorescence intensity in fixed cells following Mitosox treatment and found that there is a striking increase in fluorescence in the infected cells (Figure 2A). Interestingly, we found strong Mitosox staining of the nucleoli of many of these cells (magnified inset, Figure 2A). The nucleolus is considered to be an important sensor of cellular stress and is known to respond to oxidative stress signals [35]. Oxidative stress is known to modulate ribosomal RNA synthesis and hence influence the nucleolus [36]. We then asked the question whether oxidative stress contributes to SOD1 down regulation or oxidative stress is the result of SOD1 down regulation. To distinguish between the two possibilities, we carried out a time course analysis of ROS accumulation in infected cells (0, 2, 4, 6, 12 and 24 h). Our data demonstrates that ROS accumulation occurs as early as 12 h post infection with a strong increase over the uninfected cells at 24 h (Figure 2B and C). Next, in order to directly address the possibility that accumulation of ROS contributes to SOD1 down regulation, we treated cells with an antioxidant, N-acetyl Cysteine (NAC). Increasing concentrations of NAC strongly increased SOD1 protein levels in infected cells (Figure 2D) thus demonstrating that SOD1 down regulation was the result of increasing ROS levels after MP12 infection. Collectively, our data provides evidence that cells experience strong oxidative stress at earlier time points following infection by MP12 virus and this contributes to SOD1 protein down regulation.

### TNFα is up regulated in MP12 infected cells

**Figure 3:**
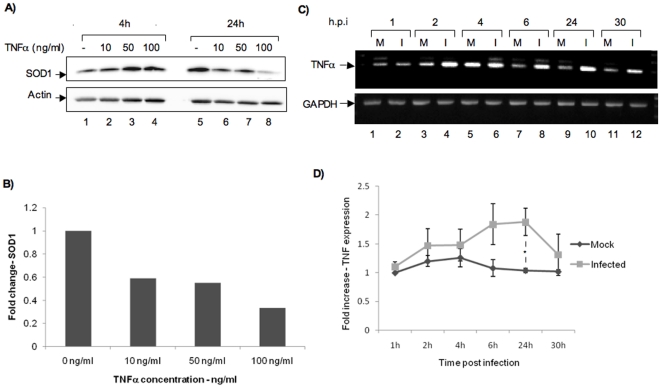
Regulation of SOD1 expression by TNFα. A) 10^6^ HSAECs were maintained in serum-minus media for 48 h. Twenty four h later, they were treated with 10, 50 and 100 ng/ml of TNFα. Total cell extracts were obtained at 4 and 24 h post addition of TNFα. The extracts were run on 4–20% Tris glycine gels and transferred on to nitrocellulose membranes. The membranes were probed with anti-SOD1 antibody. Actin was used as a loading control. B) The band intensities for all SOD1 bands were calculated using the quantity one software and Bio-Rad imaging system. All quantifications represented included normalization to actin band intensity. Quantification of SOD1 is shown for the 24-h time point and is the average of two independent experiments. C) Total RNA was extracted from MP12 infected (I) and uninfected control cells (M) at 1, 2, 4, 6, 24 and 30 h post infection. RT-PCR was performed with primers to TNFα. GAPDH was used as a control. D) Quantification of fold differences in TNFα expression between uninfected control cells and MP12-infected cells over the indicated time course. * indicates p = 0.01. Data comprises results from three experiments.

It was recently shown that in U937 human myeloid leukemia cells, increased exposure to TNFα caused a down regulation of SOD1 [37]. We wished to determine if increase in TNFα could contribute to lowered SOD1 protein levels in MP12 infected cells. To first evaluate if treatment of HSAECs with TNFα could lead to down regulation of SOD1, we examined endogenous levels of SOD1 after 4 and 24 h of exposure to increasing concentrations of exogenous TNFα. As seen in Figure 3A, at the 24-h time point, even a low concentration of TNFα reduces the endogenous SOD1 protein level to 60% of that in untreated cells. Increasing TNFα concentration further down regulates SOD1 protein levels (Figure 3A and B). The observed reduction in endogenous SOD1 following a 24 h treatment with TNFα is in agreement with the published results for U937 cells [37]. We then directly determined if viral infection of HSAECs caused up regulation of endogenous TNFα. We performed RT-PCRs with TNFα specific primers at 1, 2, 4, 6, 24 and 30 h post infection and observed up regulation of TNFα expression 24 h after infection (Figure 3C and D). Taken together, our data suggests that an increase in TNFα level may cause down regulation of SOD1 in HSAECs and that MP12 infection causes a modest up regulation of TNFα gene transcription. We had also determined by ELISA and Reverse Phase protein MicroArray (RPMA) that there was an increase in TNFα protein expression following MP12 infection (data not shown).

### Effect of depletion of SOD1 on cell cycle progression during conditions of cellular stress

**Figure 4:**
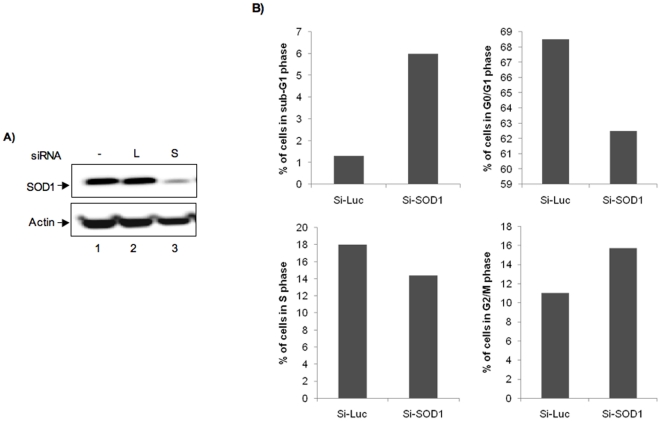
Depletion of SOD1 and effect on apoptosis. A) 10^6^ HSAECs were transfected with siRNAs against either SOD 1 (si-SOD) or luciferase (si-Luc). After 96 h, total cell extracts were made and western blots carried out using anti-SOD1 antibodies. Actin was used as a loading control. L stands for Si-Luc and S stands for Si-SOD1. B) SOD1 depleted cells and control cells (Si-Luc transfected cells) were infected with MP12 strain of RVFV (MOI of 3). Twenty four hours after infection, cells were harvested for flow cytometry analysis, stained with propidium iodide and analyzed for cell cycle progression. Percentages of cells in various stages of the cell cycle were determined and the fold differences between SOD1-depleted cells and control cells were calculated. Data is represented as average of two independent experiments.

We next evaluated the effect of lowered SOD1 level during a viral infection on apoptosis. SOD1 has been linked to the regulation of apoptosis [38]. Cell cycle progression and apoptosis have implications for the success of a virus in establishing a productive infection and viruses have evolved special means to control cell cycle progression [39], [40]. We depleted SOD1 utilizing SMARTpool siRNAs (si-SOD). As a control, HSAECs were transfected with siRNAs to luciferase (si-Luc). The cells were maintained in serum-free medium for 96 h when endogenous SOD1 was about 90% depleted (Figure 4A). We then infected the siRNA-treated cells with MP12. Strikingly, we were able to observe strong cytopathic effect in the SOD1-depleted cells as early as 24 h post infection (data not shown) suggesting that a combination of lowered SOD1 levels and a viral infection could push cells faster into apoptosis. To affirm this, the cells were trypsinized and stained with propidium iodide containing staining solution and analyzed by flow cytometry. The analysis revealed that in SOD1-depleted cells, the percentage of cells in apoptosis was almost seven times greater than the control cells at 24 h (Figure 4B). This coincided with a decrease in the G1 population in the si-SOD transfected cells (Figure 4B). We also evaluated apoptosis in SOD1 depleted cells that were not infected with virus and found that the fraction of cells in apoptosis was significantly less (1.5 times greater than control cells; data not shown). Collectively, these results indicate that depletion of endogenous SOD1 makes cells more susceptible to apoptosis during MP12 infection.

### Oxidative stress due to MP12 infection activates the p38 MAPK stress response in HSAECs

**Figure 5:**
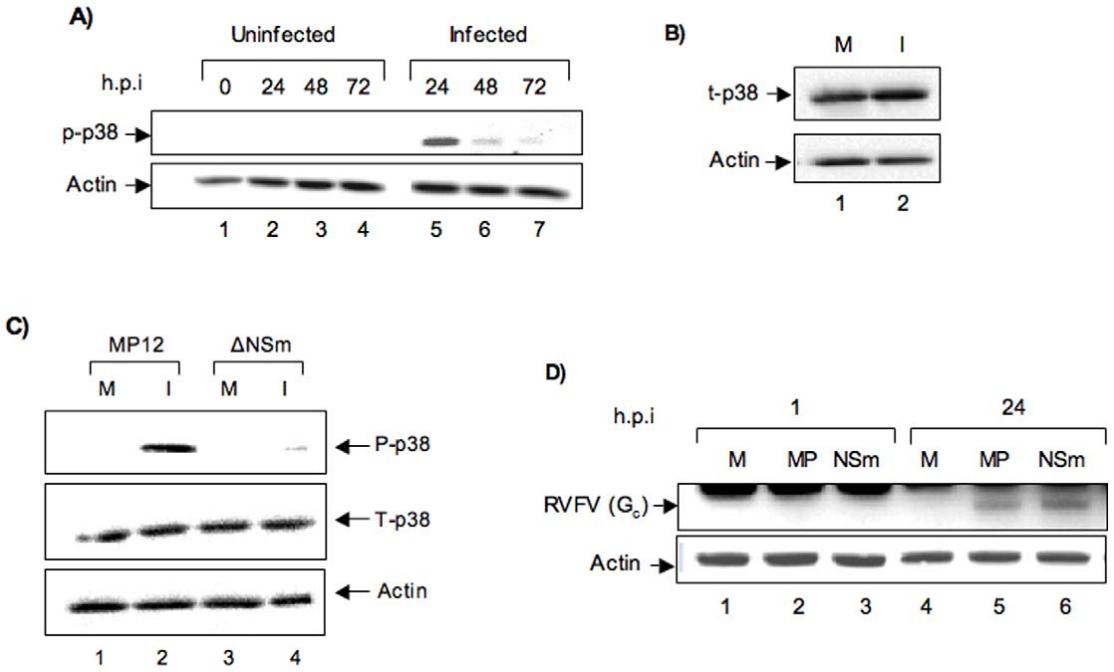
Phosphorylation of p38 MAPK following MP12 infection. A) 10^6^ HSAECs were infected with MP12 (MOI of 3). Cell extracts were obtained at 0, 24, 48 and 72 h post infection and analyzed by western blot with anti-phospho-p38 antibody. Actin was used as loading control. B) Total p38 (t-p38) levels in MP12 infected cells were compared with that of control cells at the 24-h time point by western blot with antibody to total p38 MAPK. Actin was used as loading control. C) Phosphorylation status of p38 MAPK in ΔNSm mutant virus infected cells and MP12 infected cells was determined by western blot analysis of cells infected with the NSm mutant strain (ΔNSm) and MP12 (MOI of 3). D) Comparable infection of HSAECs by MP12 virus and the ΔNSm mutant virus was determined by western blot of infected extracts obtained 24 h post infection with anti-RVFV antibody. M refers to mock-infected control cells, MP refers to MP12-infected cells and NSm refers to ΔNSm mutant virus infected cells.

Earlier studies have revealed that SOD1 is involved in the regulation of cellular cytoskeleton and siRNA-mediated depletion of SOD1 caused alterations in actin cytoskeleton in neuroblastoma cell lines [41]. As a compensatory measure, activation of the p38 MAPK pathway was observed. It was demonstrated that the activation of this stress response pathway was necessary to counter the cytoskeletal damage that occurred due to an oxidative burst observed under conditions of reduced SOD1. We reasoned that the down regulation of SOD1 (Figure 1A and B) could cause the host to activate the p38 MAPK pathway. Down regulation of SOD1 has been linked to increased phosphorylation of p38 MAPK in disease states like ALS. We next checked the phosphorylation status of p38 MAPK to determine if its activation was increased under these conditions. Western blot analysis carried out on MP12-infected cells revealed strong increase in phosphorylation of p38 MAPK at 24 h post infection (Figure 5A). We had previously demonstrated activation of p38 MAPK following MP12 infection even at lower MOIs of infection [28]. There was no significant difference in total p38 MAPK levels at the 24 h time point between mock-infected and MP12 infected cells (Figure 5B). This suggests that the increase in phosphorylation of p38 MAPK is not dependent on increased expression of p38 MAPK. Interestingly, when HSAECs were infected with a NSm mutant virus (MP12ΔNSm), similar high level of phosphorylation of p38 MAPK was not observed (Figure 5C). We confirmed similar levels of infection with the MP12 virus and the NSm mutant (Figure 5D) to rule out the possibility of lower infectivity by the NSm mutant virus. Collectively, our data suggest that down-regulation of SOD1 and a strong oxidative stress condition elicits the p38 MAPK response in HSAECs. Interestingly, our data also suggest that the viral anti-apoptotic protein NSm may play a role in the activation of p38 MAPK.

### Down regulation of SOD1 and phosphorylation of p38 MAPK in HepG2 and 293T cells

**Figure 6:**
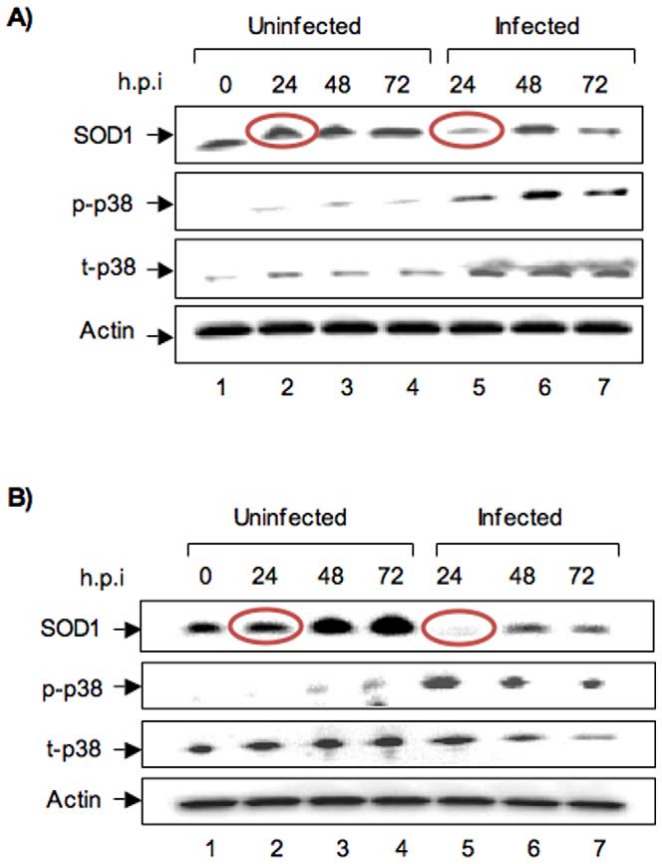
Effects of MP12 infection on SOD1 and p38 MAPK in HepG2 and 293T cells. A) 10^6^ HepG2 cells were infected with MP12 virus (MOI of 3) and extracts were obtained at 24, 48 and 72 h post infection. Western blots were carried out with antibodies to SOD1, phospho-p38 MAPK (p-p38), Total p38 MAPK (t-p38) and RVFV (Gc). B) 10^6^ 293T cells were infected with MP12 virus and analyzed by western blots as described above.

In order to determine if similar effects of infection are observed in different cell types, we infected HepG2 and 293T cells with MP12 virus (MOI of 3). We chose these cell types because HepG2 cells are liver cells and liver is a prominently affected organ in RVF. 293T cells were utilized in a recent study that screened small molecule inhibitors of RVFV and were shown to be significantly infected by RVFV [42]. Viral infection was confirmed in all cases by western blots for viral protein (data not shown). Following infection of HepG2s with MP12, we obtained cell extracts at 24, 48 and 72 h post infection. We carried out western blot analysis to determine if there is a change in the SOD1 levels in these cells. We found that similar to what we observed with the HSAECs, HepG2s showed lowered SOD1 levels at earlier time points following infection (Figure 6A, red circles). Similar to our observation in HSAECs, we also saw increased phosphorylation of p38 MAPK that peaked at 48 h post infection. Total p38 MAPK displayed only a marginal increase at all time points in the infected sample when compared to the control sample suggesting that, similar to our observations in HSAECs, the increase in phosphorylated p38 MAPK was not dependent on increased expression. Our analysis of 293T cells followed this general trend, in that, there was an early down regulation of SOD1 (Figure 6B, red circles). Interestingly, we also observed a prolonged activation of p38 MAPK as the phosphorylated form was observed up to 72 h post infection. Total p38 MAPK remained largely unchanged. Over all, the data from multiple cell types suggest that early down regulation of SOD1 and activation of p38 MAPK may be common phenomena following infection by RVFV.

### Infection by ZH501 strain of RVFV elicits similar responses in human cells as a MP12 infection

**Figure 7:**
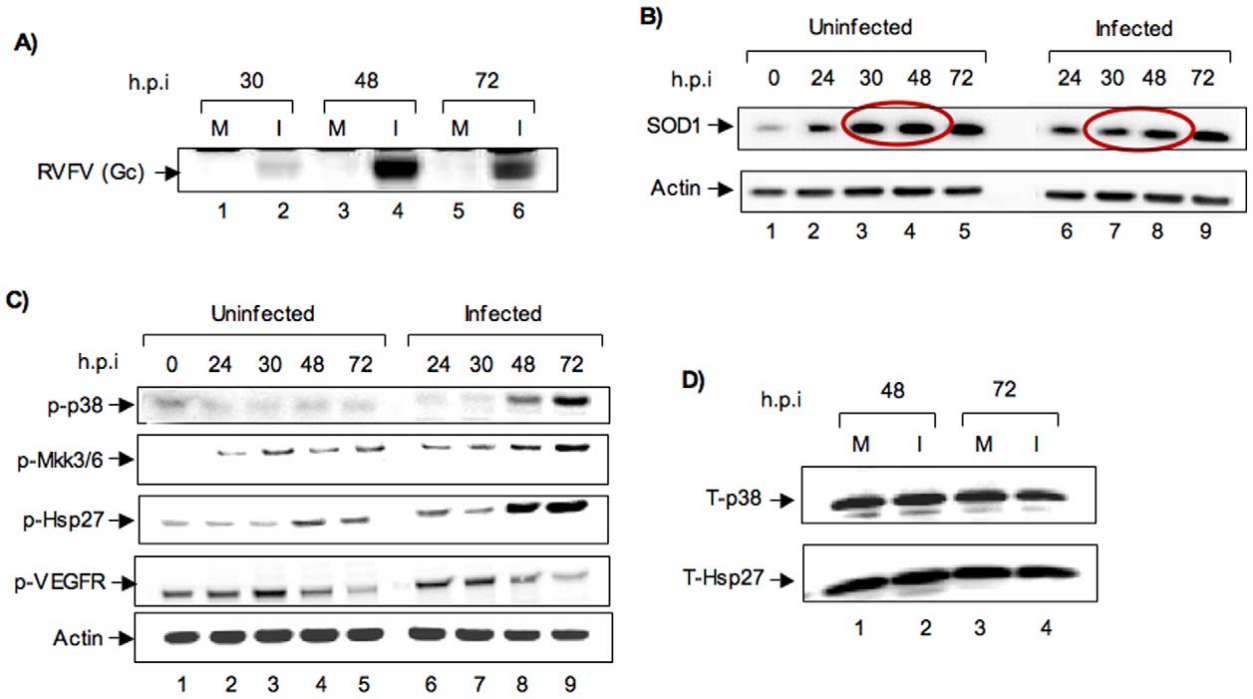
Effects of infection of HSAECs by ZH501 strain of RVFV. A) 10^6^ HSAECs were infected with the ZH501 strain of RVFV. Western blots were carried out using cell extracts obtained at 30, 48 and 72 h post infection for expression of viral proteins using anti-RVFV antibodies. B) Extracts of uninfected control cells and infected cells were resolved on SDS gels and western blots performed with anti-SOD1 antibody. Actin was used as a loading control. C) The same cell extracts used in B) were used for analysis of phosphorylated forms of VEGF-receptor, MKK3/6, p38 MAPK, and Hsp27 with specific antibodies. Actin was a loading control. D) Cell extracts obtained 48 and 72 h post infection were analyzed by western blot using antibodies to total p38 MAPK (t-p38) and Hsp27 (t-Hsp27) proteins. All data are representative of at least two independent experiments.

We then asked whether similar host responses observed due to MP12 infection can be seen when human cells were infected with the pathogenic ZH501 strain of RVFV (MOI of 0.002). HSAECs were infected with ZH501 strain and extracts were analyzed at multiple time points following infection by western blots. ZH501 infections were carried out at a lower MOI than the MP12 infections because of the strong cytopathogenecity of the virulent strain that is observed in comparison with the MP12 strain. Our results demonstrated that SOD1 levels were reduced at 24 and 30 h following infection (Figure 7B). When we evaluated the activation of the p38 MAPK pathway, we observed that p38 MAPK and its upstream kinase MKK3/6 were strongly phosphorylated between 48 and 72 h post infection. The difference in the time of activation of p38 between the MP12 strain and the ZH501 strain may be possibly due to difference in the MOI employed and differences in the inherent pathogenecities of the two strains. We also analyzed the activation status of Hsp27, a chaperone that is a downstream target of activated p38 MAPK and saw that the molecule was phosphorylated (Figure 7C). One of the upstream activators of the p38 MAPK cascade is the VEGF receptor. Our experiments did not reveal any significant increase in phosphorylation of the VEGF receptor. There was however, no change in the total protein levels of p38 MAPK and Hsp27 under these conditions (Figure 7D). Collectively, our data demonstrate that following exposure to ZH501 strain of RVFV, human cells undergo similar alterations in SOD1 protein levels and elicit similar responses as observed in the case of a MP12 infection.

## Discussion

Rift valley fever is a fast spreading zoonotic disease that affects humans and cattle causing enormous health and economic burden. While MP12 is currently under consideration for a vaccine candidate [43], there is a lack of safe, effective therapeutics for curbing viral multiplication and increasing survival of the infected host. Knowledge of the host pathophysiology during the infectious process can contribute to the development of novel therapeutics.

Oxidative stress is widely recognized as being an important contributor to disease states in multiple metabolic disorders and infectious diseases. In case of Dengue viral infections, it has been recently reported that DENV2 induces oxidative stress in mosquito cells and the infected cells survive by modulating their cellular antioxidant machinery [22]. Similarly, infections by Hepatitis B and C virus cause extensive oxidative stress that plays a significant role in liver pathology [23], [24]. We have determined that RVFV infection causes striking oxidative stress in infected cells (Figure 2A and B). One possible down stream effect of an increase in ROS following infection is a consistent reduction in the levels of SOD1, the most abundant cytoplasmic cellular antioxidant enzyme (Figure 1B, C). We observe this reduction in SOD1 following MP12 infection in several cell types (Figure 6) suggesting that oxidative stress could be a common phenomenon in various cells associated with RVFV infection. Extensive studies of multiple neurodegenerative disorders including ALS, Parkinsons and Alzheimer's disease have implicated oxidative stress as being involved in disease progression [44], [45], [46]. Particularly with ALS, functionally inactive SOD1 has been strongly implicated in disease in cell culture and animal models [47]. Restoring the cellular oxidative balance has been shown to ameliorate the effects of oxidative stress, thus emphasizing the therapeutic implication of this observation [48]. Interestingly, use of antioxidants have shown efficacy in reducing viral titers in some cases [49], [50], [51].

Our attempts to distinguish between a transcriptional and post-transcriptional mechanism for SOD1 protein down regulation suggested that it is likely to be post-transcriptional in nature. While ROS may contribute to protein instability in general, our analysis with alternate proteins such as SOD2 (Figure 1F) does not demonstrate a similar down regulation as SOD1 suggesting that the mechanism is likely to be specific to SOD1. One possible explanation for a post-transcriptional mechanism is involvement of cellular microRNAs (miRNAs). In fact, in silico analysis reveals that there are three potential miRNAs that could base-pair with the 3′UTR of SOD1 mRNA. If this mechanism is in fact true, it would be a novel means of RVFV mediated modulation of the host environment.

A recent article demonstrated that in U937 cells, increasing exposure to TNFα caused a down regulation in SOD1 expression [37]. Our experiments identified up regulation of TNFα gene expression in MP12 infected cells at time frames that correlated with the observed down regulation of SOD1 expression (Figure 3). We have confirmed increase of TNFα protein expression by alternate methods including ELISA and Reverse Phase protein MicroArray (RPMA) following MP12 infection of human cells (data not shown). Our data therefore suggests that up regulated cytokines may contribute to alterations in the oxidative balance of infected cells at early stages of infection. It is interesting to note that the viral protein NSs is known to down regulate interferon expression while we observe an increase in the expression of TNFα suggesting that there may be differential regulation of various proteins at different time points in multiple cell types due to RVFV infection.

Our observation that depletion of SOD1 caused an increase in the apoptotic population in SOD1 depleted cells than in control cells (Figure 4) suggested that the cells may activate stress responses under such conditions. It was reported that in neuroblastoma cell lines, depletion of SOD1 caused early cytoskeletal alterations in the cells that ultimately activates pro-survival pathways [41]. The report shows that activation of Hsp27 occurs via the p38 MAPK cascade and that this activation is crucial to the survival of the cell. We evaluated the activation status of the p38 MAPK cascade and observed that MP12 infection caused activation of p38 MAPK (Figure 5A). Treatment of cells with antioxidants was not sufficient to alleviate the activation of p38 suggesting that increased phosphorylation of p38 in our case is not only because of increased oxidative stress. Activation of p38 MAPK is commonly observed in many viral infections. For instance, acute infection by alpha-viruses causes phosphorylation and intracellular translocation of Hsp27 and activation of p38 [52]. In the case of cytomegalovirus, multiple components of the p38 MAPK pathway are strongly phosphorylated correlating with a progress of infection [53]. During HSV1 infection, it was demonstrated that Hsp27, in response to activation by p38 MAPK, is translocated to distinct sub-nuclear compartments called VICE (virus induced chaperone enriched) that are enriched in many of the heat shock proteins, polyubiquinated proteins and components of the proteasome machinery [54]. Along these lines, we observed that the viral anti-apoptotic protein NSm may play a role in the activation of the p38 MAPK response (Figure 5B). Viral mutants that lack NSm produce much larger plaques due to earlier onset of apoptosis in infected cells [14]. It would be very interesting to see if NSm has a role to play in the regulation of the pro-survival pathways in infected cells to prolong the life span of the cells.

Our experiments using alternate cell types produced comparable results to what was observed in HSAECs. While there are temporal differences in the down regulation of SOD1 among multiple cell types (Figures 1 and 6), there is a consistent down regulation at the early time points (24 h post infection) reinforcing this to be an early host event due to infection. Our studies of host response using ZH501 strain of RVFV have revealed that the pathogenic strain elicits similar responses as MP12. We have recently published an extensive phosphoproteomic study of ZH501 infected cells using RPMA that reveals multiple MAPK pathways, including the p38 pathway being activated following ZH501 infection [28]. Utilizing inhibitors of p38 MAPK, we demonstrated that the p38 pathway is an important protective host response.

Collectively, our data sheds light on some early mechanisms that are operational in the host cell following exposure to RVFV. It would be interesting to evaluate the effects of antioxidants as therapeutics to improve survival of infected cells and possibly control viral multiplication as well.
